# Effect of Common Comparators in Indirect Comparison Analysis of the Effectiveness of Different Inhaled Corticosteroids in the Treatment of Asthma

**DOI:** 10.1371/journal.pone.0120836

**Published:** 2015-03-20

**Authors:** Taro Kunitomi, Masayuki Hashiguchi, Mayumi Mochizuki

**Affiliations:** 1 Faculty of Pharmacy, Keio University, Tokyo, Japan; 2 Development and Medical Affairs Division, GlaxoSmithKline K.K., Tokyo, Japan; Mario Negri Institute for Pharmacology Research, ITALY

## Abstract

**Purpose:**

Indirect comparison (IC) and direct comparison (DC) of four inhaled corticosteroid (CS) treatments for asthma were conducted, and the factors that may influence the results of IC were investigated. Among those factors, we focused on the effect of common comparator selection in the treatment of asthma, where little control group bias or placebo effect is expected.

**Method:**

IC and DC were conducted using the change from baseline in forced expiratory volume in 1 s (FEV1(L)) as an outcome parameter. Differences between inhaled CS were evaluated to compare the results of IC and DC. As a common comparator for IC, placebo (PLB) or mometasone (MOM) was selected. Whether the results of IC are affected by the selection of a common comparator and whether the results of IC and DC are consistent were examined.

**Results:**

23 articles were identified by a literature search. Our results showed that ICs yielded results similar to DCs in the change from baseline of FEV1(L). No statistically significant difference was observed in inconsistency analysis between ICs and DCs. It was clinically and statistically confirmed that ICs with PLB and those with MOM did not differ in terms of the results of FEV1(L) analysis in this dataset.

**Conclusion:**

This study demonstrated that ICs among inhaled CS can deliver results consistent with those of DCs when using the change from baseline in FEV1(L) as an outcome parameter in asthma patients. It was also shown that using an active comparator has similar results if there is no effect of control group bias. It should be emphasized that the investigation of control group bias is a key factor in conducting relevant ICs so that an appropriate common comparator can be selected.

## Introduction

Indirect comparison (IC) analysis has recently been recognized as an alternative method for investigating the efficacy and safety of target interventions when head-to-head comparison data are not available. The number of studies reporting the results of ICs and network meta-analysis is increasing[1]. ICs are used not only in scientific investigations but also in healthcare decision making to assess the efficacy and safety of interventions. When used for healthcare decision making such as reimbursement evaluation and health technology assessment, some authorities, such as the Canadian Agency for Drugs and Technologies in Health, National Institute for Health and Care Excellence in the UK, and Institute for Quality and Efficiency in Health Care in Germany have been increasingly accepting IC results[2]. At the same time, some reports on ICs and network meta-analysis may not have sufficiently investigated the statistical methods and/or appropriateness of the datasets analyzed[3], and therefore it is necessary to establish transparent, uniform methods to assess the quality of ICs.

In response to the above, recently the International Society for Pharmacoeconomics and Outcomes Research—Academy of Managed Care Pharmacy—National Pharmaceutical Council (ISPOR-AMCP-NPC) Good Practice Task Force has proposed using a consensus-based 26-item questionnaire to help decision makers assess the relevance and credibility of ICs of treatment options and network meta-analysis to help inform healthcare decision making[2]. The 26 items are divided into the following five categories: evidence base (selection of study); analysis (statistical method); report quality and transparency; interpretation; and conflict of interest.

We previously reported an IC study of antipsychotics to investigate factors that may influence the results[4]. Control group bias was found to cause differing results between DC and IC. Typical control group bias can be observed between active-controlled and placebo (PLB)-controlled studies of mental disorders. If such bias occurs, the absolute value of improvement in the efficacy outcome parameter is usually greater in active-controlled trials than in PLB-controlled trials, and the absolute dropout rate is usually higher in the latter than in the former. In other words, the difference in the control group can lead to the inflation of outcome parameter scores in some therapeutic areas.

We also pointed that a well-defined endpoint should be used for IC analysis to obtain consistent results. At the same time, little control group bias and placebo effect is expected for inhaled corticosteroid (CS) studies in asthma because objective assessments such as spirometry measurement are commonly used for assessing the efficacy of inhaled CS, while subjective assessments are generally used for evaluating psychiatric diseases such as schizophrenia, depression, and anxiety disorders. In this paper, we not only report IC and DC results but also investigate factors that may influence the outcomes of IC to highlight points for consideration to ensure that it yields credible results. As one such factor, we focused on the effect of common comparator selection using inhaled CS studies for the treatment of asthma as an example.

## Methods

### Study selection

A literature search was conducted in PubMed and Embase, using the key words “fluticasone,” “budesonide,” “beclomethasone,” “mometasone,” “forced expiratory volume,” and “asthma.” Those four interventions were selected because inhaled CS is recommended as the first intervention for mild-to-moderate asthma patients in the Global Initiative for Asthma guidelines[5] that are widely followed in clinical practice.

The search was limited to “randomized controlled trial” and conducted in December 2013. All literature published in English from January 1990 through December 2013 was searched. After screening the search results, reports using similar doses and treatment periods ranging from 4 to 26 weeks were selected. If inhaler devices were different, for example, aerosol and dry-powder inhalers, it was first determined whether the conversion dosages were clinically equal. If they were equal, the data were included. Crossover studies were excluded from the analysis because the carry-over treatment effect may cause misleading results. The quality of the reports was evaluated based on the Jadad score[6], and those with scores of ≥3 were selected for this analysis. One of the authors (T.K.) initially selected the literature and extracted all the data. The literature was independently searched by another author (M.H.), who also independently confirmed each value.

### Outcome parameters

The primary efficacy endpoint for this analysis was the change from baseline in forced expiratory volume in 1 s (FEV1(L)) as assessed using spirometry.

### Stastistical methods

As a common comparator for ICs, PLB or mometasone (MOM) was selected. We first investigated whether the results of ICs were affected by the selection of a common comparator and then examined the results of ICs with PLB and DCs.

As described previously[4], for conducting ICs, we first carried out meta-analyses using the data reported in the literature between two assessed interventions using Review Manager software version 5. Mean difference analysis was conducted to assess the change from baseline in FEV1(L). We applied the random effect model in this study because some I^2^ values in meta-analysis suggested the existence of heterogeneity. In conducting IC for each analysis, we followed Bucher et al.’s method[7] using meta-analysis data obtained using Review Manager which included inhaled CS vs MOM or vs PLB.

$$D_{IC}=D_{1}-D_{2}$$

$$SE_{IC}=\sqrt{SE_{1}^{2}+SE_{2}^{2}}$$

where D_1_, D_2_ is the mean difference in the change from baseline in FEV1(L) obtained by meta-analysis between drug 1 or drug 2 and the common comparator; SE_1_, SE_2_ is the standard error of the mean difference in the change from baseline in FEV1(L) obtained by meta-analysis between drug 1 or drug 2 and the common comparator; D_IC_ is the mean difference in the change from baseline in FEV1(L) between drug 1 and drug 2 obtained by IC; and SE_IC_ is the standard error of the mean difference in the change from baseline in FEV1(L) between drug 1 and drug 2 obtained by IC.

The results were used to investigate statistical inconsistencies between IC and DC results or among ICs using different common comparators. The assumption of consistency can be evaluated by comparing D_DC_ and D_IC_ in a simple z-test[1]. We estimated the inconsistency in a closed loop as *D*_*inconsis*_ = *D*_*DC*_—*D*_*IC*_ (often called inconsistency factors) and its 95% confidence interval (95% CI) using $SE_{inconsis}=\sqrt{SE_{DC}^{2}+SE_{IC}^{2}}$ where D_DC_ is the mean difference obtained by DC and D_IC_ is that obtained by IC; and SE_DC_, SE_IC_ is the standard error of the mean difference in the change from baseline in FEV1(L) obtained by DC and IC, respectively. Inconsistency between ICs with MOM and PLB can be calculated using the same method. The 95% CI can be calculated as

$$D_{inconsis}\pm 1.96\sqrt{SE_{DC}^{2}+SE_{IC}^{2}}$$

and can be applied to for the statistical evaluation of whether there is consistency between IC and DC results.

## Results

### Eligible studies and characteristics

**Fig 1:**
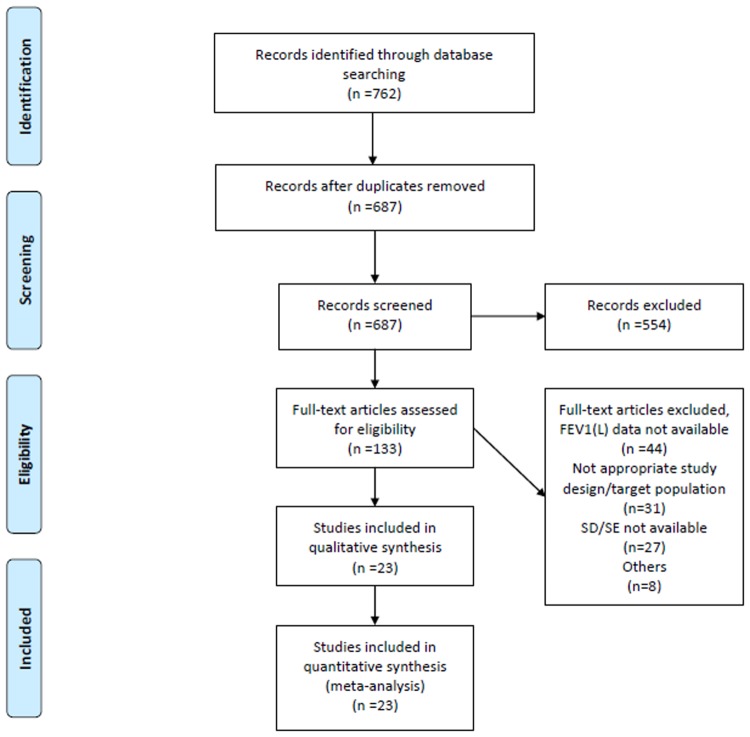
Flow diagram.

**Fig 2:**
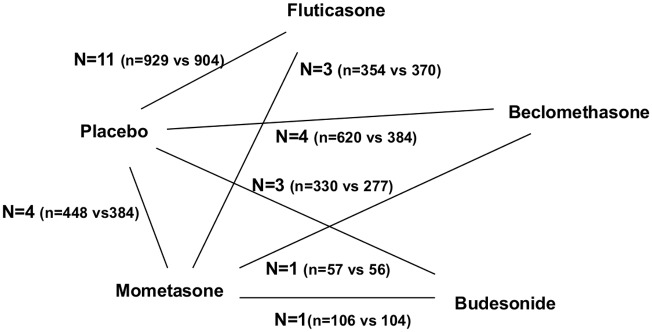
Network of studies for analysis. N: number of studies, n: number of patients.

23 studies were identified by the literature search that fulfilled the selection criteria (Table 1, Fig. 1). The majority included a PLB arm, and PLBs can be used as common comparators in various comparisons. The number of studies that compared more than one active intervention was limited, however. Fluticasone propionate (FP) was compared with MOM in three studies, beclomethasone dipropionate (BDP) was compared with MOM in one study, and budesonide (BUD) was compared with MOM in one study (Fig. 2). Those studies contributed to the formation of a closed loop for the investigation of statistical inconsistency analysis. There were no studies comparing FP vs BDP, FP vs BUD, and BDP vs BUD. As a result, MOM was selected as an active common comparator for further investigation of common comparator effects. When more than one dose was used in a report, the highest-dose arm was selected as long as it was within the US Food and Drug Administration-approved level.

**Table 1:** Details of studies included in the current IC and DC analyses.

Ref. #	Duration of study (weeks)	Intervention	*n*	Age (years) (baseline)	Duration of asthma (years)	FEV1(L) at baseline	SD (SE)	FEV1(L) change from baseline	SD (SE)
[8]	12	FP Diskhaler 250 mcg BID	184	40	13	2.46	0.05	0.16	0.04
		MOM 100 mcg BID	182	42	16	2.53	0.05	0.07	0.04
		MOM 200 mcg BID	182	42	16	2.43	0.05	0.16	0.04
		MOM 400 mcg BID	184	42	15	2.38	0.05	0.19	0.04
[9]	12	FP 250 mcg BID	81	38	na	2.44	0.07	0.42	0.05
		FP 500 mcg QD	76	37	na	2.51	0.08	0.12	0.05
		PLB	79	37	na	2.46	0.06	–0.16	0.05
[10]	12	FP 100 mcg QD	79	34	na	2.4	0.07	0.2	0.06
		FP 200 mcg QD	81	38	na	2.21	0.07	0.27	0.06
		FP 500 mcg QD	86	37	na	2.26	0.05	0.3	0.06
		PLB	84	38	na	2.22	0.06	0.11	0.06
[11]	12	FP 250 mcg BID	84	40	na	2.12	0.06	0.25	0.05
		PLB	93	38	na	2.19	0.07	–0.11	0.05
[12]	12	FP Diskus 500 mcg BID	64	32	na	2.43	0.08	0.52	0.06
		FP Diskhaler 500 mcg BID	79	34	na	2.49	0.07	0.4	0.06
		PLB	70	32	na	2.4	0.07	0.05	0.07
[13]	4	FP metered dose 88 mcg BID	23	27	na	2.91	0.13	0.27	0.07
		FP metered dose 220 mcg BID	23	21	na	2.52	0.14	0.3	0.09
		PLB	23	35	na	2.39	0.13	0	0.09
[14]	6	FP 100 mcg BID	63	40	na	2.5	0.07	0.27	0.06
		FP 500 mcg BID	69	38	na	2.36	0.07	0.42	0.06
		PLB	64	38	na	2.42	0.07	–0.19	0.08
[15]	12	FP 50 mcg	89	34	na	2.41	0.06	0.43	0.06
		FP 100 mcg	84	36	na	2.57	0.07	0.47	0.07
		FP 250 mcg	91	34	na	2.55	0.07	0.44	0.06
		PLB	78	36	na	2.41	0.08	–0.22	0.06
[16]	12	FP 100 mcg BID	119	38.3	20.6	2.425	0.6625	0.092	0.037
		PLB	118	38.1	21.4	2.352	0.6114	0.047	0.037
[17]	12	FP MDI 88 mcg BID	100	34	na	2.35	na	0.34	0.04
		FP MDI 220 mcg BID	98	34.4	na	2.5	na	0.35	0.04
		FP MDI 440 mcg BID	100	36.1	na	2.3	na	0.39	0.04
		PLB	99	31.9	na	2.4	na	0.13	0.04
[18]	12	FP MDI 250 mcg BID	113	41.9	19.93	2.14	0.585	0.106	0.041
		PLB	109	42.6	21.12	2.068	0.5222	–0.011	0.043
[19]	12	FP MDI 88 mcg BID	89	34.7	na	2.2	0.06	0.36	0.05
		PLB	87	33.2	na	2.27	0.07	0.14	0.05
[20]	12	BUD MDI 160 mcg BID	121	37.1	19.5	2.3	0.6	0.23	0.4
		PLB	122	36.1	20.8	2.4	0.7	0.03	0.44
[21]	18(6+12)	BUD DPI 400-> 200 mcg	102	35.9	19.0	2.71	0.07	0.11	0.04
		BUD DPI 200 mcg	103	38.8	18.2	2.5	0.07	0.1	0.04
		PLB	104	35.5	17.2	2.87	0.07	–0.09	0.04
[22]	12	MOM DPI 400 mcg BID	107	48	17	2.1	0.54	0.4	0.34
		FP DPI 500 mcg BID	96	49	15	2.1	0.58	0.4	0.39
[23]	12	MOM DPI 200 mcg QD	100	29.7	15.4	2.55	0.06	0.43	0.05
		PLB	95	28.6	15.9	2.64	0.06	0.16	0.05
[24]	8	MOM DPI 440 mcg QD	104	37	20	2.33	0.06	0.19	0.04
		BUD DPI 400 mcg QD	106	39	20	2.48	0.06	0.03	0.04
		PLB	51	37	20	2.5	0.08	–0.1	0.06
[25]	12	MOM DPI 100 mcg BID	185	39	na	2.49	na	0.1	0.03
		MOM DPI 200 mcg BID	176	42	na	2.52	na	0.16	0.03
		MOM DPI 400 mcg BID	188	41	na	2.54	na	0.16	0.03
		PLB	181	42	na	2.47	na	0.06	0.03
[26]	12	MOM DPI 200 mcg	79	30	16	2.58	0.07	0.27	0.06
		MOM DPI 400 mcg	74	29	17	2.64	0.07	0.41	0.06
		MOM DPI 200 mcg BID	79	32	17	2.56	0.07	0.4	0.05
		FP DPI 500 mcg BID	74	32	16	2.55	0.07	0.14	0.06
[27]	6	BDP 200 mcg BID	332	33.9	18.3	2.5	0.7	0.38	0.03
		PLB	111	33.3	21.4	2.6	0.7	0.1	0.04
[28]	12	MOM DPI 100 mcg BID	57	40	na	2.65	na	0.12	0.05
		MOM DPI 200 mcg BID	56	40	na	2.59	na	0.25	0.06
		BDP MDI 168 mcg BID	57	40	na	2.49	na	0.11	0.05
		PLB	57	42	na	2.43	na	–0.21	0.05
[29]	8	BDP MDI 252–336 mcg/day	102	37.4	20.5	2.59	0.62	0.27	0.42
		PLB	87	36.2	20.2	2.62	0.73	–0.1	0.56
[30]	26	BDP 84 mcg four times daily	129	29.9	na	2.78	0.06	0.23	0.04
		PLB	129	29.9	na	2.88	0.06	0.08	0.04

FP: fluticasone propionate, MOM: mometasone, BUD: budesonide, BDP: beclomethasone, PLB: placebo,

BID: bis in die, QD: quaque die, MDI: metered-dose inhaler, DPI: dry-powder inhaler, mcg: microgram, na: not assessed.

Safety endpoint analysis was not conducted in this study since no critical event has been reported with inhaled CS for acute-phase treatment, and limited safety information was available in this dataset.

### Indirect analysis of changes in FEV1(L) using PLB or MOM as a common comparator

**Fig 3:**
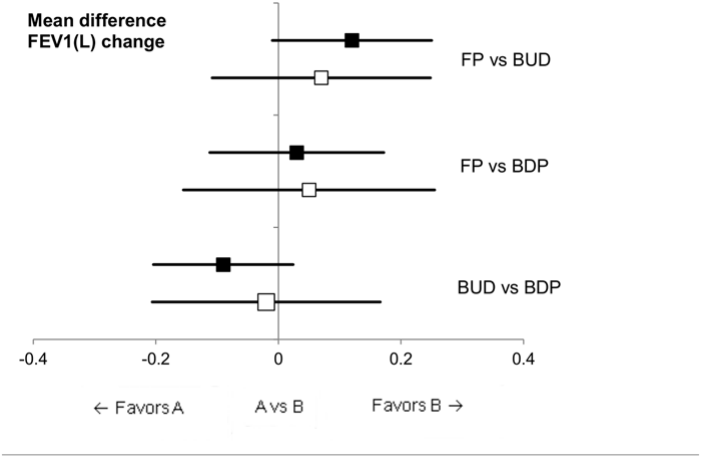
Mean difference in the change from baseline in FEV1(L): IC of PLB vs MOM. ■: indirect comparison with PLB as a common comparator; □: indirect comparison with MOM as a common comparator.

First, DCs between four inhaled CS and PLB or MOM were conducted. Subsequently, those DC data were applied for ICs between FP and BUD, FP and BDP, and BUD and BDP using PLB or MOM as a common comparator. The results of these analysis are shown in Table 2 as the mean difference (95% CI) of the change from baseline in FEV1(L). A Forest plot of those comparisons showed that there was no significant difference between ICs using PLB or MOM as a common comparator (Fig. 3). For example, in a comparison between BUD and BDP, the point estimate of mean difference was -0.09 (–0.20, 0.02) and -0.02 (–0.21, 0.17) when using PLB and MOM as the common comparator, respectively. The inconsistency was also evaluated (Table 2). The inconsistency factor with 95% CI in each comparison was FP vs BUD, 0.05 (–0.17, 0.27); FP vs BDP, –0.02 (–0.27, 0.23); and BUD vs BDP, –0.07 (–0.29, 0.15). None of the loops yielded a large value for the inconsistency statistics.

**Table 2:** Results of ICs of inhaled CS for asthma with different common comparators.

Comparison	Mean difference	(95% CI)	Inconsistency factor	(95% CI)
FP vs BUD				
IC with PLB	0.12	(–0.01, 0.25)	0.05	(–0.17, 0.27)
IC with MOM	0.07	(–0.11, 0.25)		
FP vs BDP				
IC with PLB	0.03	(–0.11, 0.17)	–0.02	(–0.27, 0.23)
IC with MOM	0.05	(–0.16, 0.26)		
BUD vs BDP				
IC with PLB	–0.09	(–0.20, 0.02)	–0.07	(–0.29, 0.15)
IC with MOM	–0.02	(–0.21, 0.17)		

### Direct and indirect analysis of changes in FEV1(L)

**Fig 4:**
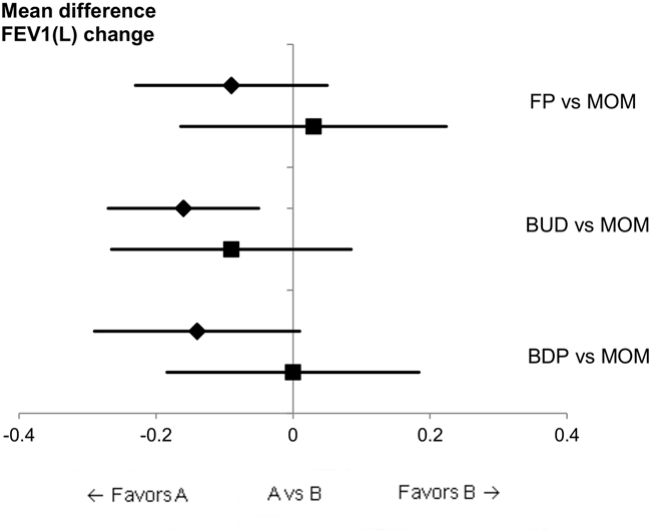
Mean difference in the change from baseline in FEV1(L): DC vs IC. ◆: direct comparison; ■: indirect comparison with PLB as a common comparator.

All head-to-head study data were used for direct analysis, allowing three DCs to be conducted (Table 3). Subsequently, the same comparisons were calculated for ICs using PLB as a common comparator. Table 3 shows the results of ICs as the mean difference (95% CI) of the change from baseline in FEV1(L) of 0.03 (–0.11, 0.17) for FP vs MOM, –0.09 (–0.27, 0.09) for BUD vs MOM, and 0.0 (–0.18, 0.18) for BDP vs MOM. The results of DCs between those interventions were -0.09 (–0.20, 0.02), –0.16 (–0.27, –0.05), and -0.14 (–0.29, 0.01), respectively. Fig. 4 shows a Forest plot of those comparisons, in which point estimates were similar between ICs with PLB and DCs. All comparisons showed that the change from baseline in FEV1(L) did not differ significantly among inhaled CS except for the DC of BUD vs MOM, which had a mean difference of -0.16 (–0.27, –0.05) in favor of BUD, while the ICs with PLB showed no statistically significant difference.

**Table 3:** Results of DC and IC of inhaled CS for asthma.

Comparison	Mean difference	(95% CI)	Inconsistency factor	(95% CI)
FP vs MOM				
DC	–0.09	(–0.20, 0.02)	–0.12	(–0.30, 0.06)
IC with PLB	0.03	(–0.11, 0.17)		
BUD vs MOM				
DC	–0.16	(–0.27, –0.05)	–0.07	(–0.31, 0.17)
IC with PLB	–0.09	(–0.27, 0.09)		
BDP vs MOM				
DC	–0.14	(–0.29, 0.01)	0.14	(–0.37, 0.09)
IC with PLB	0.0	(–0.18, 0.18)		

The results of inconsistency analysis are shown in Table 3. The inconsistency factor between DC and IC in each comparison was FP vs MOM, –0.12 (–0.30, 0.06); BUD vs MOM, –0.07 (–0.31, 0.17); and BDP vs MOM, 0.14 (–0.37, 0.09). Based on the 95% CI, there was no statistical inconsistency between DC and IC with PLB results.

### Summary of studies analyzed

#### Mean change from baseline in FEV1(L)

**Fig 5:**
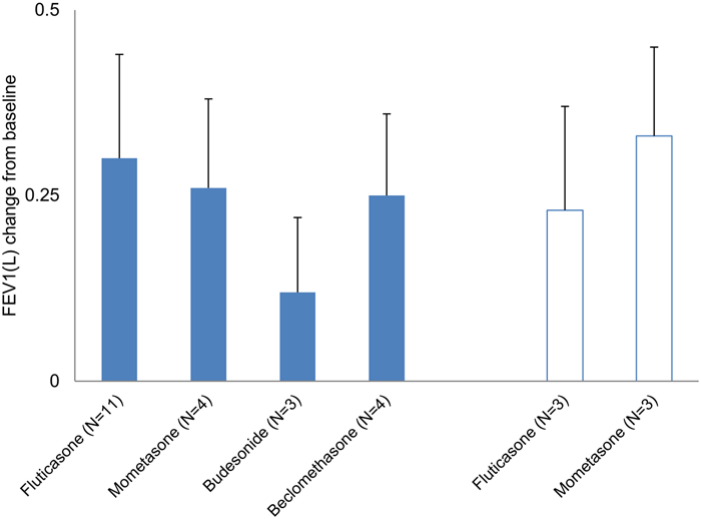
Mean FEV1(L) change in each dataset. Filled bars, placebo-controlled study; open bars, active comparator study; FEV1(L), mean change from baseline ± standard deviation.

The mean change in FEV1(L) of the inhaled CS groups in all studies are shown in Fig. 5. The results in PLB-controlled studies ranged between 0.12 and 0.3, and that in active-comparator studies between 0.23 and 0.33. When comparing the mean change in the FP and MOM groups, this result indicates that the absolute value did not vary between PLB-controlled and active-comparator studies in this dataset.

## Discussion

Inhaled CS were first launched in the 1960s and are now widely used for the treatment of asthma, recommended for both a control-based first-line treatment for mild asthma and as subsequent therapy in combination with a beta-2 agonist or leukotriene-receptor antagonist. We compared the efficacy of FP, BUD, BDP, and MOM in asthma patients using the DC and IC methods and attempted to determine the factors that may influence the DC and IC results. The effects of common comparator selection for ICs were also examined in this dataset as an example where little control group bias and placebo effect were expected. FEV1(L) change was selected as the efficacy endpoint. A previous report[4] stated that commonly defined endpoints should be selected to obtain consistent results between IC and DC. FEV1(L) is an appropriate efficacy endpoint because it is widely used, well validated, and has less placebo effect and less control group bias compared with other assessments such as patient report outcome, the PANSS for schizophrenia patients which we previously investigated, or investigators’ impression scales such as clinical global impression. Although control group bias potentially influences the results of ICs with different common comparators, this study provided useful insights on the effects of common comparators in ICs. Safety assessment data were collected as well, such as all-cause dropout rate and incidence of adverse events in this dataset. However, safety data were insufficient in the reports examined, and therefore safety parameters could not be reliably assessed.

The 23 studies included in this meta-analysis involved patients with similar demographic characteristics, such as age, duration of disease, and baseline FEV1(L) values, which may have affected the results of DC and IC. In addition, this dataset contained only five head-to-head comparisons, although 28 reports in the literature involved comparisons with PLB. The reason for this is assumed to be sponsors’ or investigators’ intent to confirm the efficacy of a treatment intervention compared with PLB rather than to show noninferiority over an active comparator. PLB-controlled studies are easier to conduct from the viewpoints of number of patients required, approval by regulatory authorities, or investigation of the comparative safety profile of an intervention. Active-comparator studies usually require more patients when a noninferiority/superiority confirmation study is designed.

No statistically significant difference was observed in inconsistency analysis between IC and DC. Clinically and statistically, ICs with PLB and MOM showed no difference in the results of FEV1(L) analysis in this dataset where no control group bias was observed. The absolute difference in FEV1(L) in point estimates ranged 0 to 0.14. A difference of 0.23 L in FEV1(L) has minimal clinical meaning in PLB-controlled trials[31].

Inconsistent results were observed in DC and IC between BUD vs MOM. DC showed a statistically significant difference in favor of MOM, whereas IC did not. We included Corren et al.’s study[24] for this DC analysis, which suggested that a disparity in baseline lung function existed between the BUD and MOM patient groups. That baseline difference was assumed to result in an absolute value disparity in FEV1(L) between the MOM and BUD groups. That study may have affected the results of the mean FEV1(L) change analysis in the BUD groups in this dataset. While the value in the mean FEV1(L) change was approximately 0.25 L in both active-comparator and PLB-controlled studies, that in the BUD group in PLB-controlled studies was 0.12 L (Fig. 5). That 0.13-L difference is believed not to be caused by control group bias but by variability due to the inclusion of the study by Corren et al. and the small number of studies in the present meta-analysis. As summarized in Figs. 3 and 4 and Tables 2 and 3, our results showed that ICs yield results similar to DCs in the change from baseline in FEV1(L).

Regarding the selection of a common comparator, Salanti et al. addressed different PLB effects in ICs using four topical fluoride treatments and two control interventions (and no treatment) in preventing dental caries in children[32]. They found that the no-treatment group and four PLB groups (i.e., toothpaste, gel, rinse, and PLB varnish) had different clinical effects although they found no statistically significant difference in consistency analysis. Salanti et al. concluded that those comparators were not exchangeable and could not be merged to conduct mixed-treatment comparisons. Our results suggest a similar point. When we use a common comparator that has a different effect, e.g., placebo effect or control bias, that effect may lead to differing results and cannot be compared even if statistical consistency is observed. It should be emphasized that clinical investigations on merging evidence should be carefully conducted. In our study, we did not observe any effect of common comparator selection in ICs, probably because we used the well-validated endpoint of FEV1(L) for assessment and investigated efficacy in asthma patients where little placebo effect is expected and the double-dummy method and/or other measures were appropriately applied to the studies included in this analysis. Similar investigations should be conducted to obtain relevant results in other ICs.

In pharmaceutical development, a PLB arm is frequently used to investigate the efficacy and safety of drugs, especially in dose-finding and early phase studies. However, it is less common in confirmatory phase 3 studies, especially when a difference in efficacy is apparent between PLB and active comparators. As described in detail previously[4], ICs can potentially be used to shorten the total development period in such situation by eliminating the step for confirmatory studies with approved drugs. Because if credible IC results can be obtained by using dose finding data with PLB or data from a trial with an active comparator, investigators can explore the efficacy and safety of a new investigational drug compared with current approved treatment. Our study shows that if the number of DC studies between active comparators is limited as in this dataset, one solution would be using PLB-controlled studies to conduct ICs if it is believed that there is little placebo effect, little control group bias in the dataset, and a well-validated endpoint is used. Under these conditions, ICs of active comparators could be expected to yield clinically meaningful results.

This report highlights the importance of common comparator selection. If the effects of both control group bias and number of DCs are limited, researchers are encouraged to use IC in a head-to-head approach because it can be expected to yield results similar to DC with fewer difficulties. One of limitations of the present study is that the number of studies using DCs and ICs is limited. Therefore further investigations and examples are necessary to clarify the importance of common comparator selection. Secondary it should be noted that selecting homogeneous population could be controversial for generalizability of the result. It can allow a good control of confounder and result as we have shown, on the other hand it might hinder generalizability of the result in more various settings. This point needs to be considered as well.

## Conclusions

This study demonstrated that IC between inhaled CS can deliver results consistent with those of DC when using the change from baseline in FEV1(L) in asthma patients. It was also shown that using active comparators has similar results when control group bias is limited. It should be emphasized that determining the degree of control group bias is a key factor in conducting relevant, appropriate IC and selecting appropriate common comparators.

## Supporting Information

S1 Fig Result of relevant direct comparison. (DOCX)

S1 PRISMA Checklist (DOC)
