# Supplementary material for: Effect of Common Comparators in Indirect Comparison Analysis of the Effectiveness of Different Inhaled Corticosteroids in the Treatment of Asthma
Source: PLoS One. 2015 Mar 20;10(3):e0120836. doi: 10.1371/journal.pone.0120836 (PMC4368804; doi:10.1371/journal.pone.0120836)

S1 Figure 1 Result of relevant direct comparison

1) Direct comparison of FP vs PLB


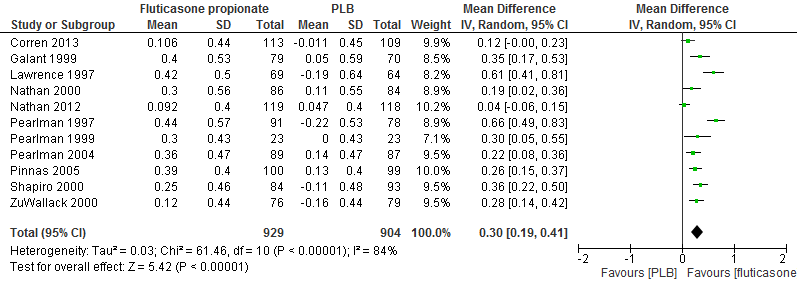


2) Direct comparison of BUD vs PLB


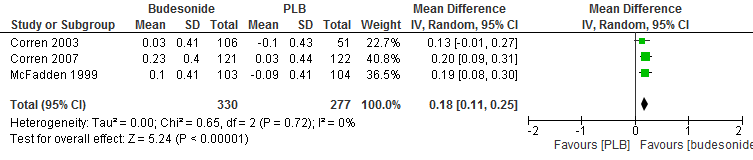


3) Direct comparison of BDP vs PLB


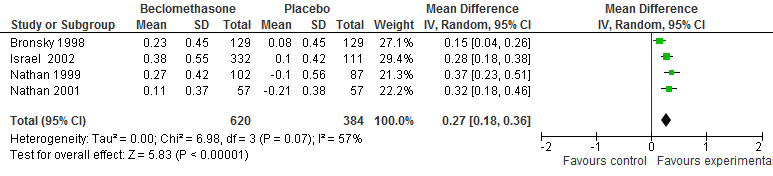


4) Direct comparison of FP vs MOM


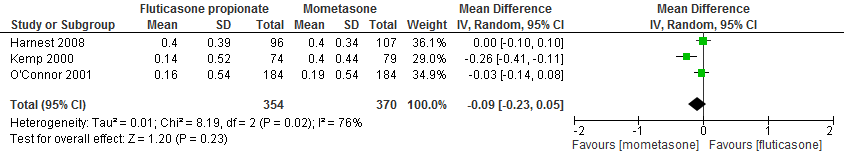


5) Direct comparison of BUD vs MOM


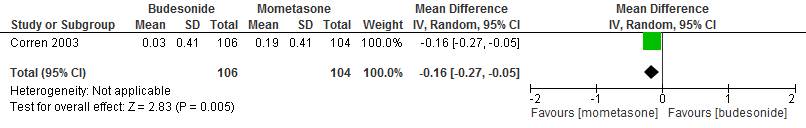


6) Direct comparison of BDP vs MOM


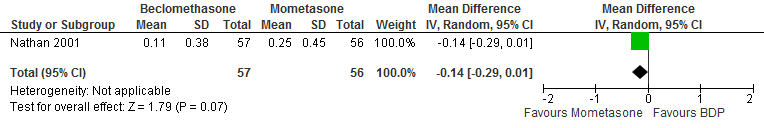

Supplement: S1 Fig — (DOCX) [file pone.0120836.s001.docx]
